# Photoluminescence Study of Gallium Nitride Thin Films Obtained by Infrared Close Space Vapor Transport

**DOI:** 10.3390/ma6031050

**Published:** 2013-03-15

**Authors:** Guillermo Santana, Osvaldo de Melo, Jorge Aguilar-Hernández, Rogelio Mendoza-Pérez, B. Marel Monroy, Adolfo Escamilla-Esquivel, Máximo López-López, Francisco de Moure, Luis A. Hernández, Gerardo Contreras-Puente

**Affiliations:** 1 Instituto de Investigaciones en Materiales, Universidad Nacional Autónoma de México, Coyoacán, México DF, C.P. 04510, Mexico; E-Mail: marel@iim.unam.mx; 2 Escuela Superior de Física y Matemáticas del IPN, Edif. 9, Unidad Profesional Adolfo López Mateos, Col. Lindavista, México DF, C.P. 07738, Mexico; E-Mails: jaguilar@esfm.ipn.mx (J.A.-H.); aescamilla@esfm.ipn.mx (A.E.-E.); jdemoure@fis.cinvestav.mx (F.M.); schwarzerengelxv@hotmail.com (L.A.H.); gscp1953@hotmail.com (G.C.-P.); 3 Facultad de Física, Universidad de La Habana, La Habana, C.P. 10400, Cuba; E-Mail: omelo@fisica.uh.cu; 4 Universidad Autónoma de la Ciudad de México, Campus San Lorenzo Tezonco, México DF, C.P. 09790, Mexico; E-Mail: kayabix@yahoo.com.mx; 5 Departamento de Física, Centro de Investigación y Estudios Avanzados del IPN, México DF, C.P. 07360, Mexico; E-Mail: mlopez@fis.cinvestav.mx

**Keywords:** solar cells, infrared-CSVT, GaN, photoluminescence, thin films

## Abstract

Photoluminescence (PL) studies in GaN thin films grown by infrared close space vapor transport (CSVT-IR) in vacuum are presented in this work. The growth of GaN thin films was done on a variety of substrates like silicon, sapphire and fused silica. Room temperature PL spectra of all the GaN films show near band-edge emission (NBE) and a broad blue and green luminescence (BL, GL), which can be seen with the naked eye in a bright room. The sample grown by infrared CSVT on the silicon substrate shows several emission peaks from 2.4 to 3.22 eV with a pronounced red shift with respect to the band gap energy. The sample grown on sapphire shows strong and broad ultraviolet emission peaks (UVL) centered at 3.19 eV and it exhibits a red shift of NBE. The PL spectrum of GaN films deposited on fused silica exhibited a unique and strong blue-green emission peak centered at 2.38 eV. The presence of yellow and green luminescence in all samples is related to native defects in the structure such as dislocations in GaN and/or the presence of amorphous phases. We analyze the material quality that can be obtained by CSVT-IR in vacuum, which is a high yield technique with simple equipment set-up, in terms of the PL results obtained in each case.

## 1. Introduction

Many semiconductor materials have been studied for their potential application in solar cells taking advantage of the possibility to perform band-gap-engineering, as well as the tailoring of optical and electrical material properties towards optimal solar-conversion performance in these devices. Among these materials, gallium nitride (GaN) has been considered as a promising candidate for thin film heterojunctions (as antireflective coating, window material, transparent emitter and back surface field material in PIN structures) and tandem solar cells due to its wide band gap (3.4 eV at 300 K), good stability at high temperature, excellent thermal conductivity and the possibility of obtaining high quality thin films with n or p type conductivity (since it is easily doped with silicon or magnesium for n or p type materials, respectively) [1,2,3,4]. GaN, generally crystallizing in the wurtzite structure (lattice constants: a = 3.189 Å, c = 5.186 Å), is a very attractive material for applications in optoelectronic devices because it has a direct band gap and it is easy to control its carrier concentration and conductivity type [5,6]. Also, the incorporation of Indium in the GaN matrix allows the band gap modulation in In*_x_*Ga*_x_*_−1_N alloys. All of these features make GaN promising for production of PIN structure (p-GaN/InGaN/n-GaN) based solar cells [7].

Many methods have been developed to deposit high quality GaN materials on different substrates. The more traditional methods are molecular beam epitaxy (MBE) and metal-organic chemical vapor deposition (MOCVD) [8,9,10]. However, these methods require very expensive equipment and have very slow growth rates. In this paper we propose a method that can be used to fabricate GaN thin films, namely, the deposition technique by infrared close space vapor transport (CSVT-IR), which has several advantages over MBE and MOCVD, such as a simple equipment set-up and high yield. This technique has been used to grow other materials such as CdTe and CdS at laboratory level and in industrial processes. Also, it can be readily scaled for mass production of polycrystalline CdTe thin film solar cells and requires only short operating times due to its fast growth rate [11].

On the order hand, photoluminescence spectroscopy (PL) is a widely used technique for qualitative study of GaN samples since it is a simple, non-destructive, contactless and effective technique. Luminescence is a very strong tool for detection and identification of point defects in semiconductors, especially in wide-band-gap varieties. A fairly complete investigation on the optical properties of wurtzite GaN on sapphire has been accomplished in different works, such as accurately measuring the GaN band-gap energy, determining the phonon modes of GaN and identifying the energy levels due to impurities or defects [12,13]. However, further investigation of GaN grown over different substrates is necessary for a better understanding of the optical properties of these thin films.

In this work we have grown GaN thin films over different substrates by the infrared CSVT technique. We have studied the dependence of the PL spectral features, such as the relative intensity and position of PL peaks, as a function of the different substrates used during the growth process. We discuss the material quality in terms of the PL analysis. We show that it is possible to obtain high quality GaN polycrystalline thin films by CSVT-IR for potential applications in solar cell devices.

## 2. Experimental Section

**Figure 1:**
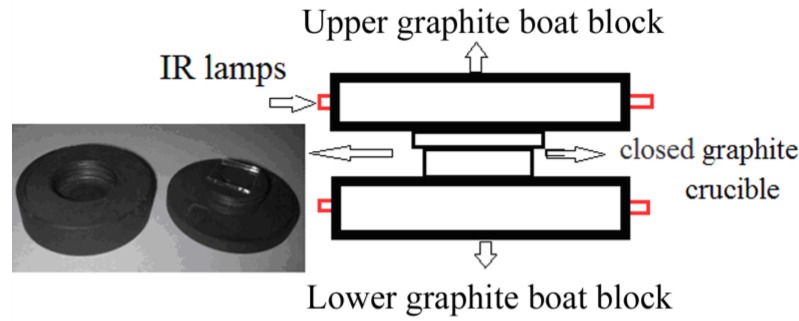
Experimental set-up scheme and photograph of the graphite crucible used in the CSVT system.

The experimental set-up includes four IR lamps, two for the upper substrate graphite block and two for the lower source graphite block, permitting the operation at high temperatures between 600 and 1000 °C, thus the name of CSVT-IR system. The CSVT sublimation system uses a graphite boat located in a heated plate which operates at around 950 °C in vacuum. To reduce as much as possible the degradation of the GaN powder source (Aldrich 99.999%) due to the very incongruent sublimation of GaN, a closed graphite crucible was used. The experimental set-up scheme is shown in Figure 1, where a photograph of the crucible is also included. It has a screw cap (left) that can be threaded onto the lower part containing the GaN source powder and the substrate (right). Typical substrate sizes were around one squared inch. Growth experiments were performed in vacuum using a pump that provides a base pressure of around 1 Pa. According to the Pressure–Temperature phase diagram of GaN [14,15], the required growth temperatures can be as high as 2400 K at N_2_ pressures of 60,000 Torr. It is impossible to decompose N_2_ vapor molecules in the growth chamber by thermal means, due to their very strong binding energy of around 9 eV/molecule. Then, assuming that the decomposition of GaN occurs following the reaction 2GaN^solid^ → 2Ga^gas^ + N_2_ it would be necessary to invoke an alternative mechanism for the dissociation of N_2_ to explain the formation of GaN by CSVT. Therefore it has been proposed that liquid Ga can catalyze the N_2_ decomposition [16]; this process is also controlled by a kinetic barrier that has been evaluated in around 3 eV [17]. Then, it could be thought that GaN formation by CSVT proceeds through a sequence of Ga liquid droplets deposition and further Ga nitridation under the high flux of N_2_ atoms. Although in our CSVT-IR system the attainable temperatures in vacuum are lower, these variables are still located in the equilibrium curve corresponding to the coexistence of phases GaN (solid) + N_2_ (gas) and Ga (metal) + N_2_ (gas), making the growth of GaN thin films feasible with this system. The source and substrate temperatures were monitored with K-type thermocouples connected to temperature controllers that regulate the switching of solid state relays to turn the IR lamps “on” and “off”, maintaining the temperature constant and uniform. The deposition was carried out on a variety of substrates like sapphire, silicon and fused silica. The silicon wafers were p-type doped with boron (ρ ~ 0.1 to 1 Ω cm) grown in the (111) orientation. Table 1 lists in detail the deposition conditions for each growth.

For structural characterization, a PANalytical XPert PRO diffractometer using Cu K_α_ radiation (λ = 1.5418 Å) was employed. Measurements were carried out with a grazing incidence of 1°, in the interval from 15° to 80° with steps of 0.03° and time steps of 5000 s. Images of the surface of the samples were acquired using a scanning electron microscope (SEM) JEOL JSM-6300 while atomic concentration measurements were obtained by energy dispersive spectroscopy (EDS) with an XFlash 5010 detector from Bruker installed in the microscope, with an acceleration voltage of 20 kV. Photoluminescence (PL) spectra were obtained using a He–Cd laser with excitation wavelength of 325 nm and output power of 16 mW, at room temperature. The sample emission was focused into an Acton SpectraPro 2500i spectrograph and detected by a photomultiplier tube. All the spectra were corrected taking into account the spectral response of the system.

**Table 1:** Deposition conditions for each growth by infrared close space vapor transport (CSVT-IR).

Sample	Substrate	Source temperature (°C)	Substrate temperature (°C)	Deposition time (min)
GaN-Za	Sapphire	950	650	10
GanN-Fs	Fused silica	950	650	10
GaN-Sip	Polished Si	950	650	10

## 3. Result and Discussion

**Figure 2:**
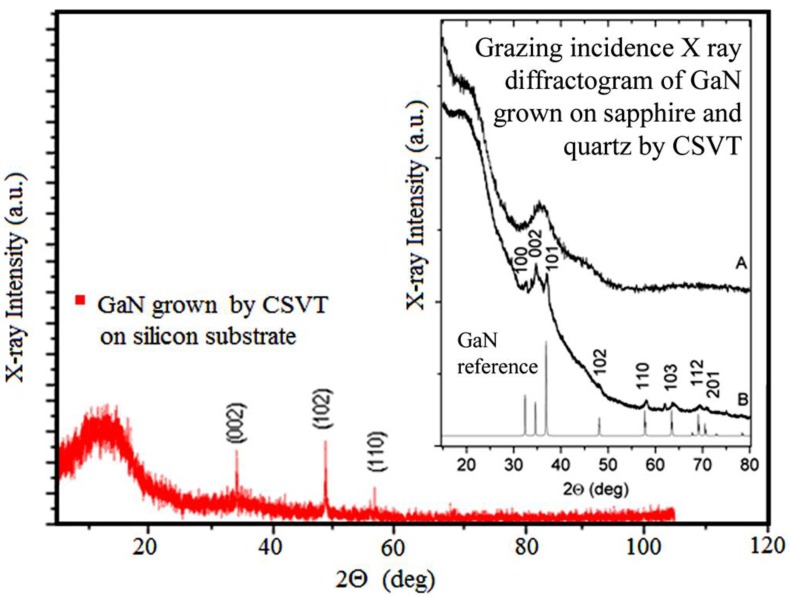
X-ray diffraction (XRD) pattern of a representative GaN sample grown on p-Si (111) substrate. The insert shows the diffractograms of samples grown on fused silica (**A**) and sapphire (**B**) and the GaN reference.

**Figure 3:**
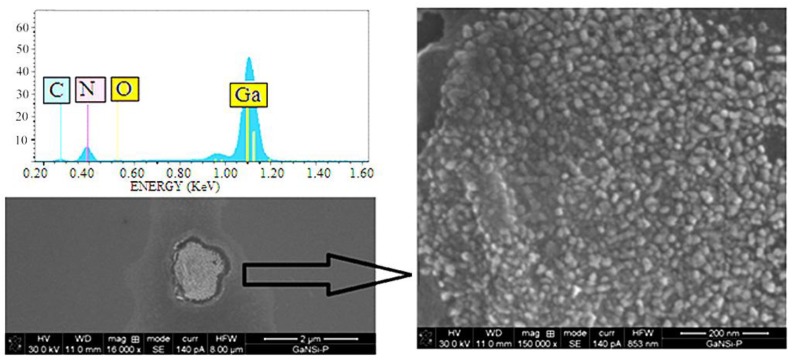
Scanning electron microscope (SEM) micrograph and energy dispersive spectroscopy (EDS) spectrum of the polycrystalline GaN thin film grown on silicon substrate.

Figure 2 shows the X-ray diffraction (XRD) curve of a representative GaN sample grown on p-Si (111) substrate. The diffraction peaks observed in Figure 2 are in good agreement with the Joint Committee for Powder Diffraction Standards (JCPDS) data for bulk GaN in the wurtzite phase. Three peaks corresponding to crystalline planes (002), (102) and (110) are located at 34.58°, 48.56° and 57.66°, respectively, demonstrating that the films can be indexed to the hexagonal GaN phase with lattice constants a¼ = 3.191 Å and c¼ = 5.192 Å [18,19]. The more intense peaks reported for wurzite GaN (PDF number 897522) are the (101) and (100) which are expected at 2θ = 36.84° and 32.39°, respectively. Since these peaks are not observed in the XRD curve, it can be presumed that GaN grains grew preferentially in the (002), (102) and (110) crystalline directions over the silicon (111) surface. A broad band at 13.80° was detected for all the samples, regardless of the substrate used. This corresponds to the presence of GaN amorphous phase. The insert in Figure 2 shows the diffractograms of samples grown on fused silica (A) and sapphire (B) substrates. In sample B, most of the peaks can be indexed as corresponding with wurzite GaN. For comparison, a reference diffractogram of GaN powder is shown. There are two small peaks that do not correspond to GaN at 2θ = 33° and 62°. They can be presumably assigned to a crystalline phase of gallium oxide. The samples grown on fused silica substrates do not present any diffraction peak and only the broad amorphous bands at 13.8° and 34° were observed. The SEM micrograph of the film grown on silicon (Figure 3) further reveals the polycrystalline nature of the films. The typical grain sizes are around 50 nm. The films thicknesses were from 50 to 60 nm in cross section measurements. Thus, growth rates over 5 nm/min can be obtained with this technique.

**Figure 4:**
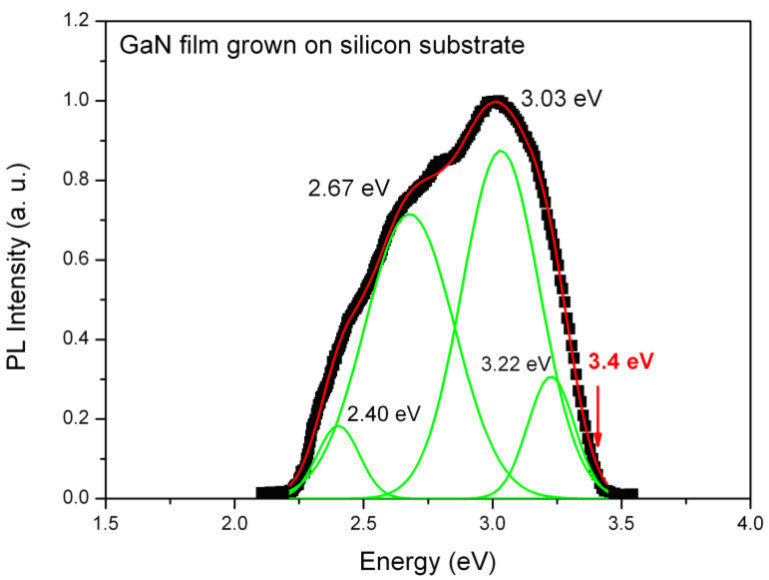
Room temperature photoluminescence spectrum for a sample grown by infrared CSVT technique on silicon substrate.

**Figure 5:**
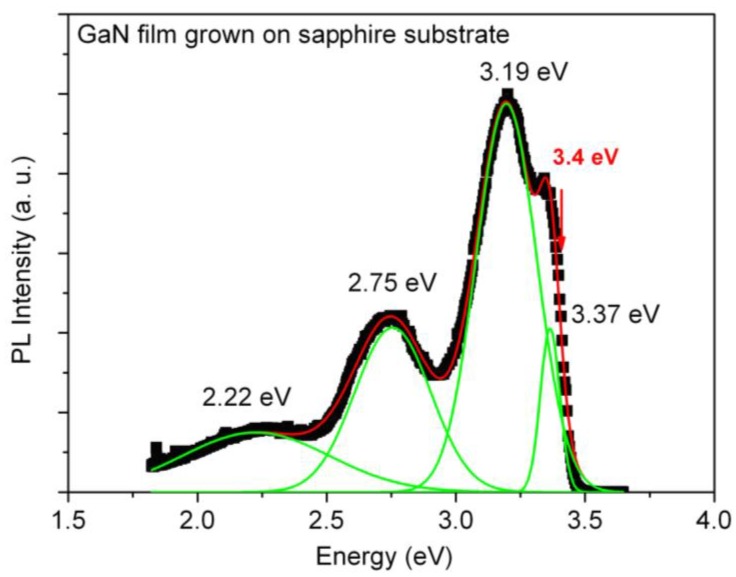
Room temperature photoluminescence spectrum for a sample grown by infrared CSVT technique on sapphire substrate.

**Figure 6:**
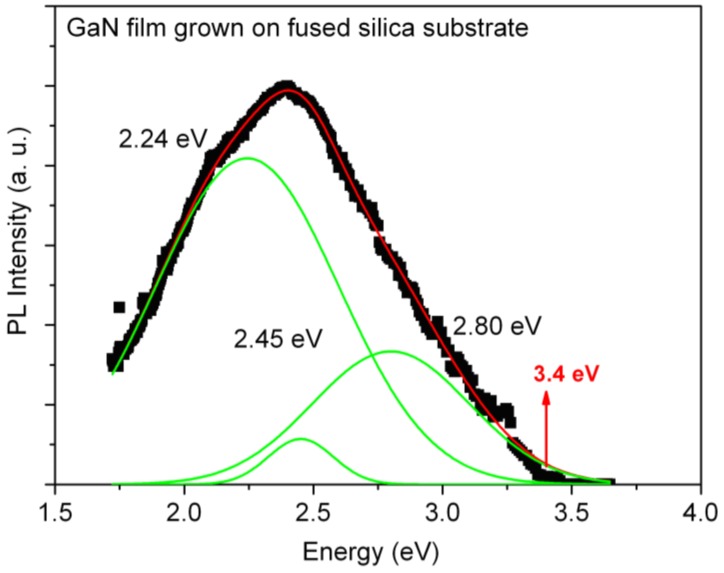
Room temperature photoluminescence spectrum for a sample grown by infrared CSVT technique on fused silica substrate.

Room temperature PL spectra of GaN films grown on different substrates are shown in Figure 4, Figure 5 and Figure 6. These graphs contain a strong near band-gap-edge emission (NBE) and a broad blue, green and yellow luminescence (BL, GL, YL), which are due to the presence of Ga and N vacancies, oxygen or deep level impurities, and amorphous phases [12,19,20]. The sample grown by infrared CSVT on the silicon substrate shows several emission peaks from 2.4 to 3.22 eV with a pronounced red shift with respect to the band gap energy (shown with a red arrow in Figure 4). A broad and intense NBE luminescence centered at around 3.0 eV and a broad shoulder luminescence at 2.67 eV in the central region can be remarkably seen in this spectrum. A deconvolution analysis was performed to interpret the possible origin of the PL bands and to evaluate the structural quality of the films. The observed band at 3.22 eV is most probably due to the direct band-to-band recombination between holes in the valence band and electrons in the conduction band of the non-stoichiometric GaN film. Another possible interpretation is that the ultraviolet luminescence (UVL) band is caused by donor-acceptor-pair (DAP) transitions from the shallow donors to the shallow acceptors [13]. At elevated temperatures, the DAP-type transitions are gradually replaced by transitions from the conduction band to the same acceptor (e-A transitions).

The rest of the peaks can be interpreted in terms of transitions due to defects and impurities introduced in the middle of the band gap. The peak at 3.03 eV may originate from the luminescence coming from the dissociation of excitons bounded to neutral donors (D°X), according to Reshchikov and Morkoc [12]. In the central region, there is broad and intense BL band at 2.67 eV associated to a luminescent center produced at dislocation edges originating from Ga and N vacancies. This is consistent with the non-stoichiometric composition in as-grown GaN thin films, which was confirmed by energy diffraction spectrum (EDS) measurements showing the films are nitrogen poor. In general, a yellow luminescence band has been reported for samples grown by MBE or MOCVD over several substrates [12]. In our case, the YL band does not appear or it is not evident for the thin films grown on silicon substrate. We believe that this band has been replaced by a green luminescence (GL) band at about 2.4 eV. The presence of the GL band in thin GaN films could be explained by the combination of gallium vacancy clusters and oxygen (or carbon) impurities denoted as *V*_Ga_–O_N_ complexes, which are preferably bound to dislocations [12,21,22]. These complexes may affect the charge state and other characteristics of the dislocation defects. The presence of this GL band is associated with an inferior quality of GaN grown over silicon substrate with respect to the material grown by traditional deposition methods.

Defects that generate visible luminescence may be increased by the use of silicon substrates. If the defects are related to the V_Ga_–O_N_ complex, the observed features can be understood as the segregation/reaction of O impurities with dislocations being induced during the growth process at high temperature, as is the case of the CSVT-IR process. From the PL analysis, we can see that GaN thin films grown on silicon substrates by CSVT-IR, do not have a stoichiometric composition and they contain a large amount of impurities as evidenced by the carbon and oxygen peaks measured by EDS in Figure 3. Nevertheless, they can be suitable for applications in hetero-structure solar cells on Si substrates, where epitaxial quality is not essential, as has been recently demonstrated [2].

In order to have more insight of the structural quality of as-grown GaN thin films grown by the CSVT-IR technique, PL measurements were used to identify the luminescence originating from dislocations as well as stacking faults of films grown over sapphire substrates. The GaN layers were grown at a substrate temperature of 650 °C and source temperature of 950 °C. The PL spectrum of this material has a sharp and strong emission peak at 3.19 eV, with a pronounced shoulder at 3.37 eV as shown in Figure 5. The 3.37 eV peak is associated to exciton recombination since the emission energy is very close to the band-gap value of GaN. The intense peak at 3.19 eV can be associated with basal-plane stacking faults [12,22], or recombination of excitons bound to prismatic stacking faults [20,21]. Another possible interpretation is that the peak is associated to DAP-type emission involving very shallow acceptors and excitons bound to structural defects, in particular, to stacking faults and *c*-axis screw dislocations. The intensity of the 3.19 eV band is related to a significant presence of stacking faults or dislocations and point defects in the samples [23].

The PL spectrum also reveals the presence of a BL at 2.75 eV and the typical YL at 2.22 eV, which are attributed to gallium and nitrogen vacancies and deep level impurities. The presence of the YL has also been observed as a result of high doping, causing carrier concentrations in the range of 10^19^ cm^−3^. However, in the present case the GaN thin films were not intentionally doped. On the other hand, several authors noted a correlation between the BL and YL band intensities and intentional carbon doping [24,25]. In our experimental setup the carbon crucibles at 950 °C can act as an unintentional doping source. Considering the fact that carbon impurities have been detected in our samples, we cannot rule out PL bands associated with carbon doping. The intensity of the 3.19 peak can also increase with C doping [26]. Furthermore, in another report a broad blue band between 2.7 and 3.0 eV, instead of the UVL band, has been observed in semi-insulating GaN:C [26]. Seager *et al.* [27] attributed this blue band to transitions from C_Ga_ donor to C_N_ acceptor. Polyakov *et al.* [28] also noted an enhancement of a blue band peaked at 3.05 eV in GaN samples that were heavily contaminated with carbon.

The PL spectrum of the GaN films deposited on fused silica is shown in Figure 6. This film exhibited a strong green-blue emission peak centered at 2.38 eV. The emission intensity was enough to be observed with the naked eye in an illuminated room. The deconvolution of the spectrum consists of three peaks centered at 2.24, 2.45, and 2.80 eV. The higher energy peak can be associated to band gap recombination in amorphous GaN (a-GaN) thin films [29]. This value agreed well with the optical band gap of a-GaN calculated from ab-initio molecular dynamics and the blue luminescence shown in the a-GaN deposited by compound source molecular beam epitaxy [29,30]. The peak at 2.45 eV can have the same origin as the GL band discussed previously for the sample grown on silicon substrate. The very intense YL band (2.24 eV) can be due to unintentional carbon doping [28] and it can also originate from trap states generated from intrinsic defects due to N vacancies, which are not so prevalent in the stoichiometric composition. This film had the worst structural quality as was expected from the use of an amorphous substrate.

In summary, it is important to note that the deposition of GaN thin films by CSVT was achieved in this work. The films were polycrystalline when grown on silicon (111) and sapphire substrates and amorphous on fused silica. The film with best structural qualities was obtained on the sapphire substrate as inferred from PL analysis. However, of special interest is the film grown on silicon substrate since it is a potential candidate for GaN/silicon heterojunction solar cells, to be used as window material or emitter in this kind of devices. Even though more studies regarding homogeneity of deposition and an optimization of the deposition process are needed, we have shown that it is possible to grow GaN thin films on this kind of substrate using a technique that can be compatible with industrial processes, as opposed to the traditional techniques.

## 4. Conclusions

Several GaN-thin film samples were processed over different substrates by the CSVT-IR technique and characterized by room-temperature photoluminescence. From the EDS analysis, we can see that GaN thin films grown by CSVT-IR, do not have a stoichiometric composition and they contain point defects such as nitrogen and gallium vacancies and the inclusion of impurities (C, O) that produce radiative transitions observed in PL experiments. The samples grown on silicon and sapphire substrates are polycrystalline, whereas the samples grown on fused silica result in amorphous GaN. The PL analysis allowed the comparative evaluation of the material quality of the films grown on different substrates. It is worth noting that PL is a simple, nondestructive technique that can provide valuable information about these materials, as we have discussed in this work. We have shown that it is possible to obtain suitable quality GaN polycrystalline thin films that are apt for applications in hetero-structure solar cells. It is very important to remark the simplicity of the CVST-IR technique, which permits to grow GaN thin films at high growth rates and it can be scaled to suit industrial processes.
